# Loss of Sustained Activity in the Ventromedial Prefrontal Cortex in Response to Repeated Stress in Individuals with Early-Life Emotional Abuse: Implications for Depression Vulnerability

**DOI:** 10.3389/fpsyg.2013.00320

**Published:** 2013-06-04

**Authors:** Lihong Wang, Natalie Paul, Steven J. Stanton, Jeffrey M. Greeson, Moria J. Smoski

**Affiliations:** ^1^Center for Biomedical Research Imaging, Tsinghua University, Beijing, China; ^2^Brain Imaging and Analysis Center, Duke University Medical Center, Durham, NC, USA; ^3^Department of Psychiatry and Behavioral Science, Duke University Medical Center, Durham, NC, USA; ^4^Center for Interdisciplinary Decision Science, Duke University, Durham, NC, USA; ^5^Department of Psychology, Oakland University, Rochester Hills, MI, USA; ^6^Duke Integrative Medicine, Duke University Medical Center, Durham, NC, USA

**Keywords:** early-life stress, repeated stress, depression vulnerability, depression resilience, fMRI, ventromedial prefrontal cortex

## Abstract

Repeated psychosocial stress in early-life has significant impact on both behavior and neural function which, together, increase vulnerability to depression. However, neural mechanisms related to repeated stress remain unclear. We hypothesize that early-life stress may result in a reduced capacity for cognitive control in response to a repeated stressor, particularly in individuals who developed maladaptive emotional processing strategies, namely trait rumination. Individuals who encountered early-life stress but have adaptive emotional processing, namely trait mindfulness, may demonstrate an opposite pattern. Using a mental arithmetic task to induce mild stress and a mindful breathing task to induce a mindful state, we tested this hypothesis by examining blood perfusion changes over time in healthy young men. We found that subjects with early-life stress, particularly emotional abuse, failed to sustain neural activation in the orbitofrontal and ventromedial prefrontal cortex (vmPFC) over time. Given that the vmPFC is known to regulate amygdala activity during emotional processing, we subsequently compared the perfusion in the vmPFC and the amygdala in depression-vulnerable (having early-life stress and high in rumination) and resilient (having early-life stress and high in mindfulness) subjects. We found that depression-vulnerable subjects had increased amygdala perfusion and reduced vmPFC perfusion during the later runs than that during the earlier stressful task runs. In contrast, depression-resilient individuals showed the reverse pattern. Our results indicate that the vmPFC of depression-vulnerable subjects may have a limited capacity to inhibit amygdala activation to repeated stress over time, whereas the vmPFC in resilient individuals may adapt to stress quickly. This pilot study warrants future investigation to clarify the stress-related neural activity pattern dynamically to identify depression vulnerability at an individual level.

## Introduction

Stress is a significant risk factor for depression and anxiety. Chronic stress can produce significant detrimental effects psychologically and physiologically (McEwen and Stellar, 1993). In response to chronic stress, some individuals develop neural habituation and adapt to the stress, but others may become sensitized to the stressor and may have prolonged or amplified neural responses over time (McEwen and Stellar, 1993). Studying neural response to a repeated stressor over time enables us to characterize neural signatures to identify depression vulnerability. The pattern of individual differences in response to a repeated stressor over time may become an important feature in differentiating vulnerability to depression and anxiety from resilience at an individual level. However, neuroimaging studies on repeated stress are rare, and the impact of repeated stress on neural circuits related to depression vulnerability remains unclear.

McEwen and Stellar (1993) proposed four types of maladaptive responses to repeated stress: (1) lack of habituation, (2) prolonged response, (3) inability to reduce reactivity, (4) inability to respond. For the first three types of responses, stronger neural, or physiological responses may be found in response to a repeated stressor compared to a single-time stressor. Studies in peripheral reactivity such as blood pressure have demonstrated this reactivity pattern (Schneider et al., 2003). However, brain regions that reflect this response pattern to repeated stress are still under investigation.

By contrast, a number of brain regions such as the ventromedial prefrontal cortex (vmPFC), ventrolateral prefrontal cortex (vlPFC), amygdala, and hippocampus can reveal maladaptive response patterns in individuals vulnerable to stress in response to repeatedly presented stressors (McEwen, 1999; Taylor et al., 2004, 2011). Dysfunction of these regions while processing emotional events are frequently reported in individuals with depression (Siegle et al., 2007; Fitzgerald et al., 2008; Wang et al., 2008), anxiety, post-traumatic stress disorder (PTSD) (Shin et al., 2004a,b), and in individuals vulnerable to depression such as those with an early-life stress history (Matsumoto et al., 2009; Pechtel and Pizzagalli, 2011). Early-life stress history refers to exposure to emotional or physical abuse, such as neglect and harsh and chaotic parenting in childhood (Bernstein et al., 2003). Both the vmPFC and vlPFC have direct projections to the amygdala (Sah et al., 2003; Holland and Gallagher, 2004) and have been found to exert a top-down, inhibitory effect on the amygdala. Typically, while regulating negative affect, healthy individuals have demonstrated higher vmPFC (Urry et al., 2006) or vlPFC (Taylor et al., 2006) activation and lower amygdala activation. A study on early-life stress (Taylor et al., 2006) found that in response to threatening cues, healthy controls showed a *negative* correlation between activation of the right vlPFC and amygdala activity, whereas participants with an early-life stress history showed a *positive* correlation between the two regions. These results suggest a possible failure to recruit the prefrontal cortex effectively for regulating emotional responses to threatening cues, and thus no reduction in amygdala activity was found in individuals with early-life stress. Therefore, it is important to further clarify the neural response pattern between the prefrontal cortex and the amygdala to repeated stressors in individuals with early-life stress.

We hypothesized that individuals with early-life stress would show an activation pattern in response to a repeated stressor that reflects long-term stress exposure, characterized by sustained amygdala hyperactivation and attenuated activation in the vmPFC or vlPFC over time due to failed recruitment of sustained activity from the prefrontal cortex to regulate amygdala response continuously.

While history of early-life stress undoubtedly is a risk factor for future depression (Wals and Verhulst, 2005), not everyone with early-life stress eventually develops depression (Charney and Manji, 2004). Individual differences in the ability to regulate stress and emotional reactions to stressful situations can modulate brain activity and peripheral physiology, with implications for vulnerability vs. resilience to depression. For example, individuals who tend to ruminate about chronic stress and negative emotions develop depression more easily in response to acute life stress (Nolen-Hoeksema, 1991). Rumination is defined as “repetitively focusing on the fact that one is depressed; on one’s symptoms of depression; and on the causes, meanings, and consequences of depressive symptoms” (Nolen-Hoeksema, 1991). It has been consistently found that rumination increases negative mood (Ward et al., 2003) and predicts the onset and relapse of depression (Nolen-Hoeksema, 1991; Nolen-Hoeksema et al., 1993; Just and Alloy, 1997). Therefore, rumination is a key cognitive trait underlying vulnerability to depression.

Some individuals with early-life stress may develop traits that enable them to be resilient (Marks et al., 2010; Binder et al., 2011; Carli et al., 2011). One such trait is mindfulness. Trait mindfulness, also known as dispositional mindfulness, refers to the self-regulation of attention as well as an orientation of openness, curiosity, and acceptance to all experiences (Bishop et al., 2004). Individuals who are high in trait mindfulness in daily life demonstrate better psychological health and lower incidence of depression (Keng et al., 2011). Therefore, it is conceivable that those who have developed trait mindfulness in spite of having an early-life stress history have a different neural activity pattern in response to a repeated stressor compared to individuals with early-life stress who have developed trait rumination.

We hypothesized that individuals with early-life stress, will show increasingly or consistently high activation in the amygdala and low or quickly reduced cognitive regulatory responses of the vmPFC following a repeatedly presented mild stressor during later relative to early trials. We predicted this activation pattern to repeated stress over time will be found particularly in individuals with early-life stress and high trait rumination, whereas an opposite pattern will be found in individuals with early-life stress and high trait mindfulness.

## Materials and Methods

### Participants

To avoid the confounding influence of menstrual cycle on stress sensitivity (Ossewaarde et al., 2010), only male participants [*n* = 19, mean (SD) age = 27.05 (7.21)] were recruited in the study. Participants were recruited from the subject registry at the Duke-UNC Brain Imaging and Analysis Center. The exclusion criteria were: (1) MRI contradictions, (2) current or history of neurological and psychiatric disorders, (3) drug abuse, and (4) current medication use. The study was approved by the Duke University Health System Institutional Review Board. All participants have signed written consent.

### Procedures

A stress induction task and a mindful breathing task were administered over two different days separated by 7–10 days. The order of the stress and mindfulness tasks was counterbalanced among the participants. Each day was composed of a pre-scan stress/mindfulness task training session and an imaging scan session. Detailed procedures can be found in our previous report (Paul et al., 2013). A number of questionnaires were administered during pre-scan session to quantify affective state [*Positive Affect and Negative Affect Scale*, PANAS (Watson et al., 1988)], state anxiety level [*Spielberger State and Trait Anxiety Inventory*, STAI-State (Spielberger et al., 1983)], self-reported stress level [*Perceived Stress Scale*, PSS (Cohen et al., 1983)], trait rumination [*Ruminative Response Scale*, RRS (Nolen-Hoeksema, 1991)], and trait mindfulness [*Five Facet Mindfulness Questionnaire*, FFMQ (Baer et al., 2006)]. Early-life stress was assessed using the Childhood Trauma Questionnaire (CTQ) (Bernstein and Fink, 1998), which contains five subscales: three assessing abuse (emotional, physical, and sexual) and two assessing neglect (emotional and physical). Early-life stress experiences were defined as either having at least three out the five subscales with a score >6 or meeting any of the following cut-off scores (Bernstein and Fink, 1998): emotion abuse ≥9; emotion neglect ≥10; physical abuse ≥10; physical neglect ≥10; or sexual abuse ≥8.

The imaging session was composed of an anatomical scan, a resting state scan, and four pairs of stress (or mindful breathing) task and an emotional go/no-go (EGNG) task runs. Using this design, we previously investigated the effect of stress and mindfulness inductions on successful inhibition to negative vs. to neutral pictures during the EGNG task (Paul et al., 2013). In this report, we focus on the blood perfusion changes during while subjects performed the stress task and the mindful breathing task. Changes in heart rate, respiration rate, and cortisol level were measured during the stress and mindful breathing tasks to objectively evaluate stress level. After completing each stress or mindful breathing task run, self-ratings of stress were also obtained immediately using a likert scale (ranged from 0 to 4, from not stressful at all to very stressful). Salivary cortisol levels were measured at the beginning, middle, and end of the scan session. All imaging scans were completed in the late afternoon to obtain low and stable cortisol levels, when individuals are more responsive to stimulation (Jansen et al., 1998) during these hours. Caffeine, smoking, and exercise were prohibited 2 h prior to scanning.

### Experimental design

#### Stress induction task

**Figure 1:**
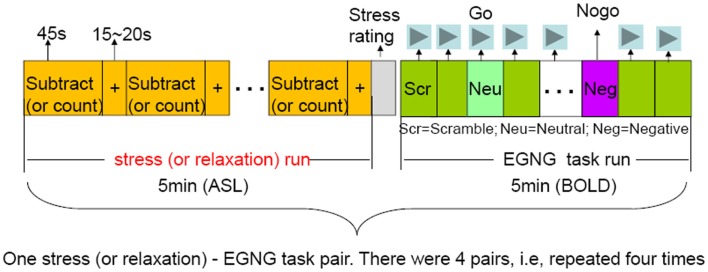
**Overview of the study design**. There were four pairs of the stressful-EGNG tasks (also the same for the mindful-EGNG tasks). In this report, we focused our analysis on the stress (and mindful) task period and compared the repetition effect of stressful (and mindful) condition on perfusion changes over time (Run 1 vs. Run 4).

We used a mental arithmetic paradigm (Soufer et al., 1998; Wang et al., 2005) to induce stress similar to the Trier Social Stress Test (Kirschbaum et al., 1993). Participants were given a four-digit starting number and a two-digit integer (presented for 5 s) at the beginning of a run. Participants had to subtract a two-digit integer from the starting number and subtract continuously. The subtraction was temporarily paused every 45 s when a fixation cross was presented, and continued when the fixation cross disappeared. Participants reported the final subtraction value at the completion of a run. Each run started with a different number and participants subtracted a different integer from the starting number during each run. Each run lasted 5 min. At the end of each run, subjects rated their stress level using a 1–4 analog bar (with 1 being not stressful at all and 4 being very stressful) (Figure 1).

#### Mindful breathing task

During the pre-scan session of the mindful breathing task day, participants were trained to: (1) focus their attention on the bodily sensations of breathing and count breaths from 1 to 10, (2) notice their mind wander and return to counting when mind wandering happens, and (3) simply return attention to breathing without getting frustrated when their mind wanders. These instructions mirror a commonly used mindfulness meditation practice (Hanh, 1976). Participants were instructed to follow these instructions during the mindful breathing task. For both stress and mindful tasks, participants paused when a fixation cross was displayed on the screen (Figure 1).

We validated the stress and mindful breathing task in our previous study. Subjects rated the stress induction task as more stressful, had higher cortisol level, faster heat beat, and respiratory rate during the stress task across all runs than during the mindful breathing task (Paul et al., 2013). In this study, we investigated whether modulated moderated the effect of repeated mental stress.

### Biochemical and physiological measures

To validate the stress and mindful tasks, peripheral biochemical and physical measures were collected including salivary cortisol level, heart rate, and respiration rate. Participants were given a Salivette (Sarstedt AG & Co., Germany) to collect salivary right before, in the middle, and right after the whole scan session. Salivary cortisol levels were assessed with solid-phase Coat-A-Count ^125^I radioimmunoassays for Cortisol (TKCO) provided by Siemens Healthcare Diagnostics (Los Angeles, CA, USA) following our previous work (Schultheiss and Stanton, 2009; Stanton et al., 2009). Heart rate and respiration rate were continuously monitored during scanning using a pulse oximeter and a chest belt, respectively (Biopac Systems, Goleta, CA, USA).

### Image acquisition and analysis

A 3.0 T GE MR750 scanner was used to acquire images at the Duke-UNC Brain Imaging and Analysis Center. After acquiring a T1-weighted SPGR anatomical image (matrix = 256 × 256 × 180, 1 mm^3^) axially parallel to the anterior and posterior commissures (AC-PC) line, we acquired perfusion images during a 5-min resting state and stress/mindful breathing tasks using an arterial spin labeling (ASL) sequence. A modified FAIR with spiral out sequence using a spatially selective inversion pulse and the QUIPSSII saturation pulses sequence (Wong et al., 1998) was used to allow quantitative determination of perfusion. A delay of 1 s was inserted between the end of the labeling pulse and image acquisition to reduce transit artifact. Acquisition parameters were: repetition time (TR) = 4 s, echo time (TE) = 3.2 ms, TI1 = 600 ms, TI2 = 1600 ms, and flip angle = 90°. Thirty-four slices (64 × 64 × 34 matrix; ∼3.5 mm^3^ voxel size) were acquired from inferior to superior in a sequential order. BOLD images were acquired during the emotional go/no-go task.

The analysis was carried out using FEAT-perfusion fMRI analysis (MRI Expert Analysis Tool Version 5.98), part of the FSL analysis package (FMRIB Software Library)^1^. We conducted full perfusion signal modeling^2^, where three explanatory variables (EVs) were modeled: the BOLD signal, the (constant-height) tag-control difference, and the activation component of the tag-control signal (formed by multiplying the first two EVs together). Thus, the activation component of the tag-control signal is our study interest. The following standard preprocessing steps were taken: removal of non-brain signal outside the head using the Brain Extraction Tool (BET), slice-time correction, coregistration, motion correction, normalization, spatial smoothing (5 mm FWHM), and high-pass filtering (1/60 Hz).

Given that the primary goal of the study is to investigate the repetition effect of stress on perfusion changes over time and the modification effect of early-life stress, we focused our data analysis on between-run differences within subjects (i.e., the first run vs. the last run). Calculating the perfusion change from Run 1 to Run 4 is the way we modeled change over time, and in particular, response patterns to repeated stress. An increase in perfusion from Run 1 to Run 4 (i.e., Run 4–Run 1) demonstrates either neural sensitization in regions related to affective processing or more effective recruitment of resources in a brain region related to cognitive control, and a decrease from Run 1 to Run 4 demonstrates neural habituation or less effective recruitment of resources in a brain region related to cognitive control. The results were also confirmed by using the early two runs (Run 1 and Run 2) vs. the later two runs (Run 3 and Run 4). The between-run differences were computed using a fixed effect model for each subject under both stressful task condition and mindful breathing task condition. For the group level analysis, we first examined significant perfusion between-run difference in the group during the stress and mindful tasks separately using random effect model (FLAME1) to investigate which regions demonstrated a habituation effect over the course of the stress task and the mindfulness task across all participants. Then, to understand whether the habituation effect was stress or mindfulness specific, we examined differences in neural adaptation patterns between the two tasks. We compared the difference in perfusion of Run 1 vs. Run 4 between the stressful and mindful task condition using paired *t*-test with a random effects analysis.

To investigate the modification effect of early-life stress on habituation in response to repeated stress over time, we then conducted regression analyses to examine the association between perfusion changes from Run 1 to Run 4 with the measurements of early-life stress. Given the theory that early-life stress is associated with depression in adulthood, we first investigated the relationship between the total CTQ score and CTQ subcomponent scores with trait rumination (RRS) and trait mindfulness (FFMQ) as well as each facet of FFMQ using simple linear (total score of measurement) and multiple linear regression analysis (subcomponents). The total or subscores of CTQ which showed significant correlation (*p* < 0.05) with RRS or FFMQ were used to do the whole-brain voxel-wise analysis with perfusion changes from Run 1 to Run 4. For all imaging-related analyses, significance was determined using a voxel significance level of *z* > 2.3, with a whole-brain-corrected cluster significance threshold of *p* < 0.05.

Finally, to further understand the perfusion pattern in response to the repeated stressor across time related to depression vulnerability and resilience, we also subsequently identified individuals with early-life stress who possibly were vulnerable or resilient to depression using the following criteria: (1) vulnerable (*n* = 5), having early-life stress experiences, RRS ≥ 40, FFMQ < 150, and (2) Resilient (*n* = 5), having early-life stress experiences, RRS < 40, FFMQ ≥ 150. Individuals who did not have early-life stress experience were defined as Neutral (*n* = 6). The psychological measures for each of the groups were shown in Table 1. There were three participants who had missing RRS and FFMQ data. Because of the small sample size of the Vulnerable, Resilient, and Neutral groups, we investigated the perfusion changes using Region-of-Interest (ROI) analysis. The ROIs were identified by extracting the significant clusters from the regression analyses in the third level analysis. The mean signal strength with each ROI for each run in each subject was calculated.

**Table 1:** **The Neuropsychological measures for the three groups, group resilient to depression, the group vulnerable to depression, and the group neutral to depression that were defined based on the RRS and FFMQ scores**.

No. of subjects	Resilient	Vulnerable	Neutral
	
	5	5	6
RRS	26.4 (3.2)	43.6 (3.0)	28.5 (4.0)
FFMQ	152.2 (3.5)	125.2 (11.9)	115.3 (16.5)
Total CTQ	28.6 (3.3)	32.2 (3.3)	29.8 (6.5)
*Emotional abuse*	5.2 (0.4)	8 (1.4)	7 (2.8)
*Physical abuse*	5.8 (1.3)	5.6 (0.9)	5.5 (0.8)
*Sexual abuse*	5 (0)	5.4 (0.9)	5 (0)
*Emotional neglect*	7 (1.6)	7.8 (2.9)	6.8 (2.6)
*Physical neglect*	5.6 (0.9)	5.4 (0.5)	5.5 (0.8)

*Italics are used when referring to the subscales of the CTQ (Childhood Trauma Questionnaire)*.

## Results

### Behavioral results

#### The negative association of early-life stress with trait mindfulness

There was not a significant correlation between RRS and either the CTQ total score or the score of subcomponents. However, multiple regression analysis showed that, among the CTQ subcomponents, emotional abuse was inversely associated with non-reactivity, one of the five facets of the mindfulness on the FFMQ (*t* = 2.23, *p* = 0.05), meaning that individuals with more severe emotional abuse during childhood had lower trait non-reactivity. Trait non-reactivity is the ability to step back from one’s experiences without becoming overly engrossed in them (Baer et al., 2006), which we previously found having protective effect from depression vulnerability (Paul et al., 2013). Therefore, emotional abuse seemed to be an important risk factor for depression vulnerability, and the score of emotion abuse was used to our following neuroimaging data analysis.

### Neuroimaging results

#### The habituation to the repeated stressor and repeated mindful task

**Figure 2:**
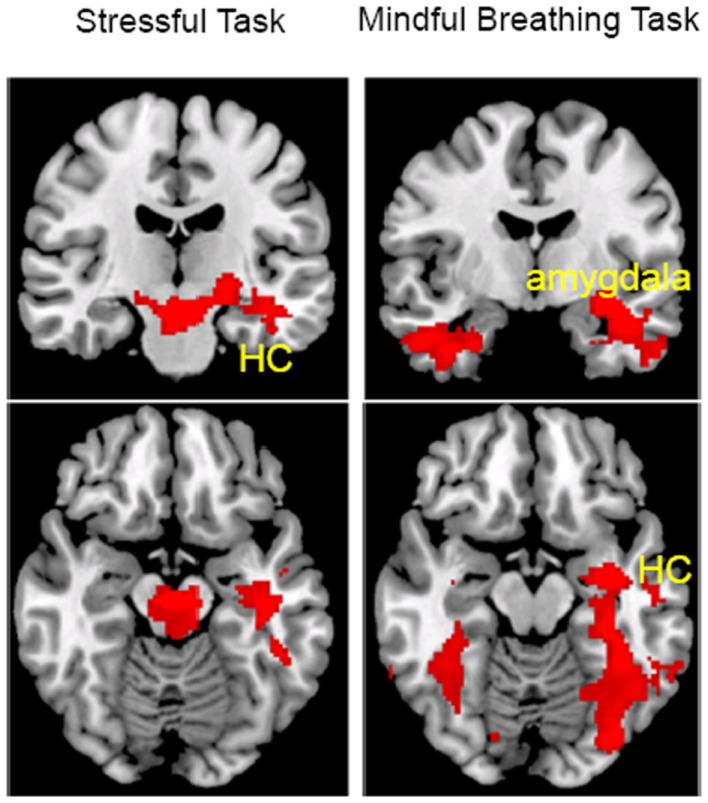
**The main effect of task repetition (Run 1–Run 4) on perfusion during the stress (left) and mindful breathing (right) tasks (voxel-wise analysis, *Z* > 2.3, *p* < 0.05 cluster correction)**. HC, hippocampus cortex.

First, we investigated regions that showed a habituation effect, i.e., regions that showed perfusion decrease from Run 1 to Run 4 (Run 1–Run 4) under the stress and mindful breathing conditions separately across all participants. The voxel-wise analyses revealed that when a stressor was presented repeatedly (Figure 2; Table 2), the perfusion was significantly decreased in the left middle and superior temporal cortex from the first to the last run (i.e., Run 1–Run 4) suggesting there was a habituation effect in these regions under the stressful condition. During the repeated mindful breathing task (Figure 2; Table 2), the perfusion was significantly decreased in the left amygdala, hippocampus, fusiform gyrus, and bilateral thalamus, therefore, there was a habituation effect in these regions during the mindfulness induction. In a direct comparison of the stress task and mindful breathing task, however, we did not find significant differences in perfusion comparing the Run 1–Run 4 contrast between the 2 days.

**Table 2:** **Regions that revealed habituation effect (Run 1–Run 4) of the stressful task and the mindful breathing task (those in italic are subclusters)**.

Region	Hemisphere	Cluster size	Peak voxel (*x*)	Peak voxel (*y*)	Peak voxel (*z*)	Maximum *Z*-value
**HABITUATION EFFECT OF THE STRESSFUL TASK**						
Cerebellum crus I	R	13957	28	−80	−22	4.36
*amygdala*	L		−*28*	−*6*	−*14*	*3.74*
*hippocampus*	L		−*32*	−*26*	−*11*	*3.01*
*temporal pole*	L		−*42*	−*12*	−*30*	*4*
	R		*40*	*0*	−*36*	*3.92*
*cerebellum vermis*	L		−*6*	−*49*	−*23*	*3.28*
*fusiform gyrus*	L		−*28*	−*56*	−*6*	*3.52*
	R		*36*	−*36*	*19*	*2.87*
Lateral parietal cortex	R	1801	32	−70	54	3.47
**HABITUATION EFFECT OF THE MINDFUL BREATHING TASK**						
Midbrain	L	1862	−6	−16	−16	4.7
*midbrain*	*R*		*6*	−*10*	−*12*	*3.7*
*pons*	*R*		*2*	−*20*	−*24*	*4.25*
*thalamus*	*L*		−*20*	−*20*	−*10*	*3.71*
*hippocampus*	*L*		−*40*	−*20*	−*16*	*3.73*

#### The association of early-life stress with less habituation to the repeated stressor

**Figure 3:**
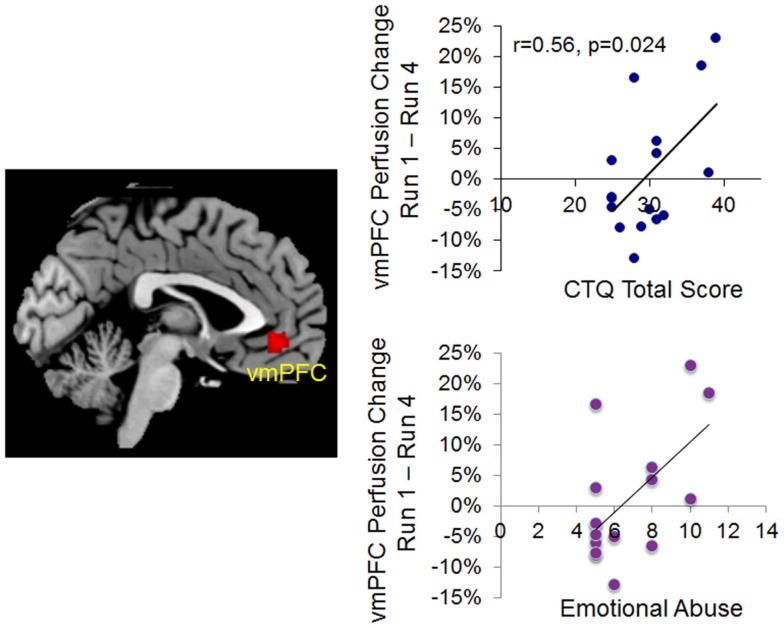
**Left, voxel-wise analysis on the correlation between emotional abuse score and the perfusion changes from Run 1 to Run 4 during repeated stress (voxel-wise analysis, *z* > 2.3, *p* < 0.05 cluster correction)**. Right, correlation plots based on ROI analyses confirming the significant correlation between the perfusion changes from Run 1 to Run 4 during repeated stress and emotional abuse score (lower plot) and Childhood Trauma Questionnaire (CTQ) Total Score (upper plot).

Given that only the emotional abuse subscore of the child trauma score was significantly correlated with mindfulness (negatively), we used the emotional abuse subscore to compute the regression analyses with the perfusion change from Run 1 to Run 4. The whole-brain voxel-wise analysis revealed that the emotional abuse subscore was significantly correlated with a reduction in perfusion level from Run 1 to Run 4 in the right orbitofrontal cortex and the vmPFC (Figure 3; Table 3) during the stress task. Taking the significant cluster of vmPFC as a ROI, we confirmed that the significant correlation between the reduction of perfusion from Run 1 to Run 4 and emotional abuse in childhood (*r*_14_ = 0.59, *p* = 0.016, for double confirm purpose) was not due to outliers (see the lower plot in Figure 3). The *post hoc* ROI analysis showed that the perfusion reduction from Run 1 to Run 4 during the stressful condition was also significantly correlated with the CTQ total score (*r*_14_ = 0.56, *p* = 0.024, Figure 3). A similar association was not found in response to the repeated mindful breathing task, indicating that reported early-life stress was uniquely associated with a habituation response in the vmPFC perfusion under acute stress conditions in the laboratory.

**Table 3:** **Regions that revealed significant correlation between perfusion reduction during repeated stress and emotional abuse (those in italic are subclusters)**.

Region	Hemisphere	Cluster size	Peak voxel (*x*)	Peak voxel (*y*)	Peak voxel (*z*)	Maximum *Z*-value
Orbital frontal cortex	R	1909	36	54	−10	4.68
*Orbital frontal cortex*	*R*		*34*	*60*	−*4*	*3.84*
*Ventromedial prefrontal cortex*	*M*		−*1*	*45*	−*6*	*3.25*

#### Habituation effect in depression-vulnerable vs. resilient individuals – subsequent ROI analyses

We further performed an exploratory investigation on the perfusion pattern in the vmPFC and in the amygdala in response to the repeated stressor in depression-vulnerable and resilient individuals. The vmPFC ROI was defined using voxels that revealed significant correlation between emotional abuse and the reduction of Run 1–Run 4 perfusion, and the amygdala ROI was defined using voxels showing significant habituation effect during the mindful breathing task. Only ROI analyses were conducted due to the small number of subjects who met the vulnerable or resilient criteria. We focused on the vmPFC and amygdala regions because of the well-known association of these two regions with early trauma (Taylor et al., 2006; Hart and Rubia, 2012) and the inverse relationship between the two regions in emotion regulation.

**Figure 4:**
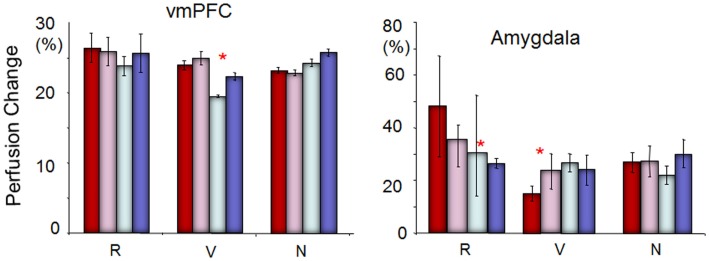
**The perfusion changing pattern in the vmPFC and amygdala to repeated stress across runs among individuals resilient to depression (*R*), vulnerable to depression (*V*), and neutral (*N*, not resilient and not vulnerable)**. Red = Run 1; pink = Run 2; silver = Run 3; blue = Run 4. The perfusion level in the amygdala was increased from Run 2 to Run 4 relative to Run 1, whereas the perfusion level in the vmPFC was decreased from Run 3 to Run 4 relative to Run 1 for individuals vulnerable to depression. On the other hand, depression-resilient individuals showed a linear decrease in perfusion level from Run 1 to Run 4, whereas the perfusion level of the vmPFC did not change significantly across the four runs. ^*^Indicates significant difference between the first two runs with the last two runs in ROI analyses (paired *t-*tests, *p* < 0.05).

As the bar graph shown in Figure 4, ROI analyses on the vmPFC and amygdala revealed an inverse relationship. For individuals vulnerable to depression, the perfusion level in the amygdala increased from Run 2 to Run 4 relative to Run 1, whereas the perfusion level in the vmPFC was decreased from Run 3 to Run 4 relative to Run 1. Interestingly, depression-resilient individuals showed a linear decrease in perfusion level from Run 1 to Run 4 in the amygdala, whereas the perfusion level of the vmPFC did not change significantly across the four runs. By contrast, individuals who did not experience childhood trauma (*N* in Figure 4) revealed a sustained activity perfusion level in the vmPFC when they were repeatedly exposure to stress and sustained activation in the amygdala.

## Discussion

The current study investigated the impact of early-life stress on blood flow over time during a repeated stress induction task and during a repeated mindful breathing task. We found a inverse correlation between emotional abuse and non-reactivity (a subcomponent of mindfulness) which is consistent with previous finding that childhood emotional abuse and neglect proved more predictive of adult depression than childhood sexual or physical abuse (Powers et al., 2009). Our voxel-wise regression analysis revealed a clear association between childhood emotional abuse and reduced orbitofrontal and vmPFC perfusion over time when participants were exposed to stress repeatedly. The orbitofrontal and vmPFC sends projections to the amygdala, and is known to regulate amygdala activity (Price, 2005; Urry et al., 2006). Therefore, we speculate that a lack of sustained orbitofrontal and vmPFC activity, coupled with amygdala hyperactivity, in response to a repeated stressor, might be an important neural signature of depression vulnerability.

Our interpretation was supported by our subsequent ROI analyses which confirmed that individuals who experienced early-life stress and were high in trait rumination had reduced vmPFC activity in the later compared to earlier stressful trials, individuals who experienced early-life stress but were high in trait mindfulness had sustained vmPFC activity, and individuals who did not have early-life stress had an increased vmPFC perfusion over time. The inverse relationship between the vmPFC and the amygdala has been consistently reported in the literature (Phelps et al., 2004; Harenski and Hamann, 2006; Ohira et al., 2006; Urry et al., 2006). Our results suggest that healthy adults exert effortful control over emotion via increasing the vmPFC activity when they are repeatedly exposed to a stressor. For individuals who develop stress resilience, they may regulate amygdala activity more efficiently than healthy adults without this trait resilience by sustaining the vmPFC activity without further increasing the effortful vmPFC activity. Whereas when individuals are vulnerable to stress, the vmPFC has a limited capacity for cognitive control to regulate emotional responding, and could not effectively regulate amygdala activity in the later runs. Therefore, our study suggests that a lack of sustained vmPFC activity when repeatedly exposed to stress might be an important neuroimaging marker that distinguishes stress vulnerability vs. resilience in individuals with early-life stress.

However, the interpretation is preliminary due to the small sample size for the stress vulnerable and resilient groups. Another caveat of the study is the fact that we only recruited males in the study. The literature on sex differences in stress response is mixed (Kudielka and Kirschbaum, 2005; Wang et al., 2007; Ossewaarde et al., 2010). According to Ossewaarde et al. (2010), women have different responses to stress and facial expressions during different menstrual phases. Their results also indicated general stress sensitivity during the late luteal phase, as indicated by stress-induced heart rate changes and reported negative affect. Indeed, a study by Kirschbaum et al. (1993) found that the salivary cortisol response of women in the luteal phase was similar to men, but women taking oral contraceptives or in the follicular phase demonstrated lower free cortisol responses. Wang et al. (2007) further showed that stress in men was associated with cerebral blood flow (CBF) increase in the right prefrontal cortex and CBF reduction in the left orbitofrontal cortex, whereas stress in women activated the ventral striatum, putamen, insula, and cingulate cortex. There, future studies with larger sample sizes of both males and females are needed to verify our findings. Studies include women, and either collect menstrual-cycle phase data or only examine women in the luteal phases are recommended, to minimize the effect of menstrual-cycle phase on stress reactivity and further clarify the literature on sex-related differences in stress reactivity. In addition, future studies using causal modeling are necessary to further understand whether a lack of regulatory control by the vmPFC over the amygdala is the most important regulatory pathway in depression vulnerability.

Of note, although the hippocampus is often regarded as the most vulnerable region to stress and has consistently been found damaged in patients with PTSD (Astur et al., 2006; Werner et al., 2009; Dickie et al., 2011; Thomaes et al., 2013), we did not find an association of perfusion change over time in the hippocampus with early-life stress. Instead, we found reduced perfusion in the hippocampus when repeatedly practicing mindful task suggesting a task learning effect. The reason that we did not find a significant association between the perfusion change in the hippocampus and early-life stress might be due to the fact that some individuals with early-life stress in our sample have developed resilience to stress. Our subsequent analysis with the amygdala demonstrated the pattern we would have expected with the hippocampus, and future studies in a larger sample size would clarify this hypothesis about stress and the hippocampus.

We also found decreased blood flow over time in the amygdala, fusiform gyrus, and bilateral thalamus specifically during the mindful breathing task. The reduced blood perfusion from Run 1 to Run 4 under the mindfulness condition fits well with previous knowledge in that repeated mindful breathing can result in habitation and less excitation over time in the regions related to emotional salience (amygdala, thalamus) and imagery memory (hippocampus and fusiform gyrus) (Byrne et al., 2007; Bird et al., 2012). Nevertheless, we did not find any significant difference in the perfusion pattern in the vmPFC or any other regions when comparing perfusion patterns over time between stress and mindful conditions. One explanation for this result might be that the stress task did not evoke a sufficiently strong stress response to see a statistical difference in our small sample size. Therefore, only individuals who had experienced early-life stress revealed stronger change in neural response to the repetition of such a mild stressor, suggesting that early-life stress might have a sensitized effect in these individuals. In this sense, a mild stressor might be more useful in detecting stress vulnerability. On one hand, our mental arithmetic task only involved mental effort but not negative feedback or psychological pressure, which are two important elements of mental stress (Dickerson and Kemeny, 2004). It will be informative in future studies to compare neural responses to different elements and different types of stressors.

In summary, we found an association between early-life stress and reduced perfusion level in the vmPFC in response to repetition of a mild stressor. The effect was particularly strong in individuals who had early-life stress and also developed trait rumination, but not in those who had strong trait mindfulness. Our results open an opportunity of assessing stress vulnerability and resilience by continuously monitoring the vmPFC and amygdala perfusion or activation change over time. Functional MRI typically has large variation across subjects and difficult to establish a norm range, which has limited its application clinically for individualized diagnosis. The dynamic changing pattern in brain perfusion over time found in this study is important because it relies on the changing pattern within subject across time rather than a norm value. If confirmed by future studies using larger samples of males and females, changing patterns of perfusion to repeated exposures of stress over time, could be a novel way to identify depression vulnerability at an individual level.

## Conflict of Interest Statement

The authors declare that the research was conducted in the absence of any commercial or financial relationships that could be construed as a potential conflict of interest.

## Footnotes

^1^www.fmrib.ox.ac.uk/fsl

^2^http://fsl.fmrib.ox.ac.uk/fsl/fsl4.0/feat5/perfusion.html

## References

[B1] Astur R. S. St Germain S. A. Tolin D. Ford J. Russell D. Stevens M. (2006). Hippocampus function predicts severity of post-traumatic stress disorder. Cyberpsychol. Behav. 9, 234–240 10.1089/cpb.2006.9.234 16640486

[B2] Baer R. A. Smith G. T. Hopkins J. Krietemeyer J. Toney L. (2006). Using self-report assessment methods to explore facets of mindfulness. Assessment 13, 27–45 10.1177/1073191105283504 16443717

[B3] Bernstein D. Fink P. (1998). Manual for the Childhood Trauma Questionnaire: A Retrospective Self-Report. San Antonio: Harcourt Brace & Co

[B4] Bernstein D. P. Stein J. A. Newcomb M. D. Walker E. Pogge D. Ahluvalia T. (2003). Development and validation of a brief screening version of the Childhood Trauma Questionnaire. Child Abuse Negl. 27, 169–190 10.1016/S0145-2134(02)00541-0 12615092

[B5] Binder E. Malki K. Paya-Cano J. L. Fernandes C. Aitchison K. J. Mathe A. A. (2011). Antidepressants and the resilience to early-life stress in inbred mouse strains. Pharmacogenet. Genomics 21, 779–789 10.1097/FPC.0b013e32834b3f35 22016050

[B6] Bird C. M. Bisby J. A. Burgess N. (2012). The hippocampus and spatial constraints on mental imagery. Front. Hum. Neurosci. 6:142 10.3389/fnhum.2012.00142 22629242 PMC3354615

[B7] Bishop S. R. Lau M. Shapiro S. Carlson L. Anderson N. D. Carmody J. (2004). Mindfulness: a proposed operational definition. Clin. Psychol. Sci. Pract. 11, 230–241 10.1093/clipsy/bph077

[B8] Byrne P. Becker S. Burgess N. (2007). Remembering the past and imagining the future: a neural model of spatial memory and imagery. Psychol. Rev. 114, 340–375 10.1037/0033-295X.114.2.340 17500630 PMC2678675

[B9] Carli V. Mandelli L. Zaninotto L. Roy A. Recchia L. Stoppia L. (2011). A protective genetic variant for adverse environments? The role of childhood traumas and serotonin transporter gene on resilience and depressive severity in a high-risk population. Eur. Psychiatry 26, 471–478 10.1016/j.eurpsy.2011.04.008 21684723

[B10] Charney D. S. Manji H. K. (2004). Life stress, genes, and depression: multiple pathways lead to increased risk and new opportunities for intervention. Sci. STKE 2004, re5 15039492 10.1126/stke.2252004re5

[B11] Cohen S. Kamarck T. Mermelstein R. (1983). A global measure of perceived stress. J. Health Soc. Behav. 24, 385–396 10.2307/2136404 6668417

[B12] Dickerson S. S. Kemeny M. E. (2004). Acute stressors and cortisol responses: a theoretical integration and synthesis of laboratory research. Psychol. Bull. 130, 355–391 10.1037/0033-2909.130.3.355 15122924

[B13] Dickie E. W. Brunet A. Akerib V. Armony J. L. (2011). Neural correlates of recovery from post-traumatic stress disorder: a longitudinal fMRI investigation of memory encoding. Neuropsychologia 49, 1771–1778 10.1016/j.neuropsychologia.2011.02.055 21382385

[B14] Fitzgerald P. B. Laird A. R. Maller J. Daskalakis Z. J. (2008). A meta-analytic study of changes in brain activation in depression. Hum. Brain Mapp. 29, 683–695 10.1002/hbm.20613 17598168 PMC2873772

[B15] Hanh T. N. T. M. H. (1976). The Miracle of Mindfulness: An Introduction to the Practice of Meditation. Boston: Beacon Press

[B16] Harenski C. L. Hamann S. (2006). Neural correlates of regulating negative emotions related to moral violations. Neuroimage 30, 313–324 10.1016/j.neuroimage.2005.09.034 16249098

[B17] Hart H. Rubia K. (2012). Neuroimaging of child abuse: a critical review. Front. Hum. Neurosci. 6:52 10.3389/fnhum.2012.00052 PMC3307045 22457645

[B18] Holland P. C. Gallagher M. (2004). Amygdala-frontal interactions and reward expectancy. Curr. Opin. Neurobiol. 14, 148–155 10.1016/j.conb.2004.03.007 15082318

[B19] Jansen L. M. Gispen-De Wied C. C. Gademan P. J. De Jonge R. C. Van Der Linden J. A. Kahn R. S. (1998). Blunted cortisol response to a psychosocial stressor in schizophrenia. Schizophr. Res. 33, 87–94 10.1016/S0920-9964(98)00066-8 9783348

[B20] Just N. Alloy L. B. (1997). The response styles theory of depression: tests and an extension of the theory. J. Abnorm. Psychol. 106, 221–229 10.1037/0021-843X.106.2.221 9131842

[B21] Keng S. L. Smoski M. J. Robins C. J. (2011). Effects of mindfulness on psychological health: a review of empirical studies. Clin. Psychol. Rev. 31, 1041–1056 10.1016/j.cpr.2011.04.006 21802619 PMC3679190

[B22] Kirschbaum C. Pirke K. M. Hellhammer D. H. (1993). The "Trier Social Stress Test" – a tool for investigating psychobiological stress responses in a laboratory setting. Neuropsychobiology 28, 76–81 10.1159/000119004 8255414

[B23] Kudielka B. M. Kirschbaum C. (2005). Sex differences in HPA axis responses to stress: a review. Biol. Psychol. 69, 113–132 10.1016/j.biopsycho.2004.11.009 15740829

[B24] Marks A. D. Sobanski D. J. Hine D. W. (2010). Do dispositional rumination and/or mindfulness moderate the relationship between life hassles and psychological dysfunction in adolescents? Aust. N. Z. J. Psychiatry 44, 831–838 10.3109/00048674 20815670

[B25] Matsumoto M. Yoshioka M. Togashi H. (2009). Early postnatal stress and neural circuit underlying emotional regulation. Int. Rev. Neurobiol. 85, 95–107 10.1016/S0074-7742(09)85007-1 19607963

[B26] McEwen B. S. (1999). Stress and hippocampal plasticity. Annu. Rev. Neurosci. 22, 105–122 10.1146/annurev.neuro.22.1.105 10202533

[B27] McEwen B. S. Stellar E. (1993). Stress and the individual. Mechanisms leading to disease. Arch. Intern. Med. 153, 2093–2101 10.1001/archinte.153.18.2093 8379800

[B28] Nolen-Hoeksema S. (1991). Responses to depression and their effects on the duration of depressive episodes. J. Abnorm. Psychol. 100, 569–582 10.1037/0021-843X.100.4.569 1757671

[B29] Nolen-Hoeksema S. Morrow J. Fredrickson B. L. (1993). Response styles and the duration of episodes of depressed mood. J. Abnorm. Psychol. 102, 20–28 10.1037/0021-843X.102.1.20 8436695

[B30] Ohira H. Nomura M. Ichikawa N. Isowa T. Iidaka T. Sato A. (2006). Association of neural and physiological responses during voluntary emotion suppression. Neuroimage 29, 721–733 10.1016/j.neuroimage.2005.08.047 16249100

[B31] Ossewaarde L. Hermans E. J. Van Wingen G. A. Kooijman S. C. Johansson I. M. Backstrom T. (2010). Neural mechanisms underlying changes in stress-sensitivity across the menstrual cycle. Psychoneuroendocrinology 35, 47–55 10.1016/j.psyneuen.2009.08.011 19758762

[B32] Paul N. A. Stanton S. J. Greeson J. M. Smoski M. J. Wang L. (2013). Psychological and neural mechanisms of trait mindfulness in reducing depression vulnerability. Soc. Cogn. Affect. Neurosci. 8, 56–64 10.1093/scan/nss070 22717383 PMC3541493

[B33] Pechtel P. Pizzagalli D. A. (2011). Effects of early life stress on cognitive and affective function: an integrated review of human literature. Psychopharmacology (Berl.) 214, 55–70 10.1007/s00213-010-2009-2 20865251 PMC3050094

[B34] Phelps E. A. Delgado M. R. Nearing K. I. Ledoux J. E. (2004). Extinction learning in humans: role of the amygdala and vmPFC. Neuron 43, 897–905 10.1016/j.neuron.2004.08.042 15363399

[B35] Powers A. Ressler K. J. Bradley R. G. (2009). The protective role of friendship on the effects of childhood abuse and depression. Depress. Anxiety 26, 46–53 10.1002/da.20534 18972449 PMC2629811

[B36] Price J. L. (2005). Free will versus survival: brain systems that underlie intrinsic constraints on behavior. J. Comp. Neurol. 493, 132–139 10.1002/cne.20750 16255003

[B37] Sah P. Faber E. S. Lopez De Armentia M. Power J. (2003). The amygdaloid complex: anatomy and physiology. Physiol. Rev. 83, 803–834 12843409 10.1152/physrev.00002.2003

[B38] Schneider G. M. Jacobs D. W. Gevirtz R. N. O’Connor D. T. (2003). Cardiovascular haemodynamic response to repeated mental stress in normotensive subjects at genetic risk of hypertension: evidence of enhanced reactivity, blunted adaptation, and delayed recovery. J. Hum. Hypertens. 17, 829–840 10.1038/sj.jhh.1001624 14704727

[B39] Schultheiss O. C. Stanton S. J. (2009). “Assessment of salivary hormones,” in Methods in Social Neuroscience, eds Harmon-Jones E. Beer J. S. (New York: NY: Guilford Press), 17–44

[B40] Shin L. M. Orr S. P. Carson M. A. Rauch S. L. Macklin M. L. Lasko N. B. (2004a). Regional cerebral blood flow in the amygdala and medial prefrontal cortex during traumatic imagery in male and female Vietnam veterans with PTSD. Arch. Gen. Psychiatry 61, 168–176 10.1001/archpsyc.61.2.168 14757593

[B41] Shin L. M. Shin P. S. Heckers S. Krangel T. S. Macklin M. L. Orr S. P. (2004b). Hippocampal function in posttraumatic stress disorder. Hippocampus 14, 292–300 10.1002/hipo.10183 15132428

[B42] Siegle G. J. Thompson W. Carter C. S. Steinhauer S. R. Thase M. E. (2007). Increased amygdala and decreased dorsolateral prefrontal BOLD responses in unipolar depression: related and independent features. Biol. Psychiatry 61, 198–209 10.1016/j.biopsych.2006.05.048 17027931

[B43] Soufer R. Bremner J. D. Arrighi J. A. Cohen I. Zaret B. L. Burg M. M. (1998). Cerebral cortical hyperactivation in response to mental stress in patients with coronary artery disease. Proc. Natl. Acad. Sci. U.S.A. 95, 6454–6459 10.1073/pnas.95.11.6454 9600987 PMC27794

[B44] Spielberger C. D. Gorsuch R. L. Lushene P. R. Vagg P. R. Jocobs A. G. (1983). Manual for the Statie-Trait Anxiety Inventory (Form Y): Self-Evaluation Questionnaire. Palo Alto: Consulting Psychologists Press, Inc

[B45] Stanton S. J. Beehner J. C. Saini E. K. Kuhn C. M. Labar K. S. (2009). Dominance, politics, and physiology: voters’ testosterone changes on the night of the 2008 United States presidential election. PLoS ONE 4:e7543 10.1371/journal.pone.0007543 19844583 PMC2760760

[B46] Taylor S. E. Eisenberger N. I. Saxbe D. Lehman B. J. Lieberman M. D. (2006). Neural responses to emotional stimuli are associated with childhood family stress. Biol. Psychiatry 60, 296–301 10.1016/j.biopsych.2005.09.027 16460697

[B47] Taylor S. E. Lerner J. S. Sage R. M. Lehman B. J. Seeman T. E. (2004). Early environment, emotions, responses to stress, and health. J. Pers. 72, 1365–1393 10.1111/j.1467-6494.2004.00300.x 15509286

[B48] Taylor S. E. Way B. M. Seeman T. E. (2011). Early adversity and adult health outcomes. Dev. Psychopathol. 23, 939–954 10.1017/S0954579411000411 21756443

[B49] Thomaes K. Dorrepaal E. Draijer N. De Ruiter M. B. Elzinga B. M. Sjoerds Z. (2013). Increased anterior cingulate cortex and hippocampus activation in Complex PTSD during encoding of negative words. Soc. Cogn. Affect. Neurosci. 8, 190–200 10.1093/scan/nsr084 22156722 PMC3575721

[B50] Urry H. L. Van Reekum C. M. Johnstone T. Kalin N. H. Thurow M. E. Schaefer H. S. (2006). Amygdala and ventromedial prefrontal cortex are inversely coupled during regulation of negative affect and predict the diurnal pattern of cortisol secretion among older adults. J. Neurosci. 26, 4415–4425 10.1523/JNEUROSCI.3215-05.2006 16624961 PMC6673990

[B51] Wals M. Verhulst F. (2005). Child and adolescent antecedents of adult mood disorders. Curr. Opin. Psychiatry 18, 15–19 16639178

[B52] Wang J. Korczykowski M. Rao H. Fan Y. Pluta J. Gur R. C. (2007). Gender difference in neural response to psychological stress. Soc. Cogn. Affect. Neurosci. 2, 227–239 10.1093/scan/nsm018 17873968 PMC1974871

[B53] Wang J. Rao H. Wetmore G. S. Furlan P. M. Korczykowski M. Dinges D. F. (2005). Perfusion functional MRI reveals cerebral blood flow pattern under psychological stress. Proc. Natl. Acad. Sci. U.S.A. 102, 17804–17809 10.1073/pnas.0503082102 16306271 PMC1292988

[B54] Wang L. Labar K. S. Smoski M. Rosenthal M. Z. Dolcos F. Lynch T. R. (2008). Prefrontal mechanisms for executive control over emotional distraction are altered in major depression. Psychiatry Res. 163, 143–155 10.1016/j.pscychresns.2007.10.004 18455373 PMC2553159

[B55] Ward A. Lyubomirsky S. Sousa L. Nolen-Hoeksema S. (2003). Can’t quite commit: rumination and uncertainty. Pers. Soc. Psychol. Bull. 29, 96–107 10.1177/0146167202238375 15272963

[B56] Watson D. Clark L. A. Tellegen A. (1988). Development and validation of brief measures of positive and negative affect: the PANAS scales. J. Pers. Soc. Psychol. 54, 1063–1070 10.1037/0022-3514.54.6.1063 3397865

[B57] Werner N. S. Meindl T. Engel R. R. Rosner R. Riedel M. Reiser M. (2009). Hippocampal function during associative learning in patients with posttraumatic stress disorder. J. Psychiatr. Res. 43, 309–318 10.1016/j.jpsychires.2008.03.011 18490028

[B58] Wong E. C. Buxton R. B. Frank L. R. (1998). Quantitative imaging of perfusion using a single subtraction (QUIPSS and QUIPSS II). Magn. Reson. Med. 39, 702–708 10.1002/mrm.1910390506 9581600

